# Research on Intelligent Distribution of Liquid Flow Rate in Embedded Channels for Cooling 3D Multi-Core Chips

**DOI:** 10.3390/mi13060918

**Published:** 2022-06-09

**Authors:** Jian Zhang, Zhihui Xie, Zhuoqun Lu, Penglei Li, Kun Xi

**Affiliations:** 1 College of Power Engineering, Naval University of Engineering, Wuhan 430033, China; 13251586887@163.com (J.Z.); l.zhuoqun@hotmail.com (Z.L.); lipenglei19940401@hotmail.com (P.L.); xikun_91@163.com (K.X.); 2 School of Energy and Electromechanic Engineering, Hunan University of Humanities, Science and Technology, Loudi 417000, China

**Keywords:** embedded cooling, dynamic thermal management, artificial neural network, genetic algorithm, nonuniform heat source

## Abstract

A numerical simulation model of embedded liquid microchannels for cooling 3D multi-core chips is established. For the thermal management problem when the operating power of a chip changes dynamically, an intelligent method combining BP neural network and genetic algorithm is used for distribution optimization of coolant flow under the condition with a fixed total mass flow rate. Firstly, a sample point dataset containing temperature field information is obtained by numerical calculation of convective heat transfer, and the constructed BP neural network is trained using these data. The “working condition–flow distribution–temperature” mapping relationship is predicted by the BP neural network. The genetic algorithm is further used to optimize the optimal flow distribution strategy to adapt to the dynamic change of power. Compared with the commonly used uniform flow distribution method, the intelligently optimized nonuniform flow distribution method can further reduce the temperature of the chip and improve the temperature uniformity of the chip.

## 1. Introduction

In Moore’s era, the feature size of semiconductor processes has been continuously reduced. The integration level of chips has been rapidly improved and the development trend of chips toward miniaturization, light weight, and high-density integration has become increasingly prominent. However, as the feature size of the semiconductor process gradually approaches the physical limit [1], the cost of improving the chip integration by reducing the feature size of the semiconductor process is getting higher and higher. In this context, advanced packaging technology represented by 3D stacked chips has become a new driver to continue to improve chip integration. The 3D stacked chip integrates multiple layers of chips in one package, which improves the space utilization of the chip, shortens the wafer interconnection distance, reduces the delay of the internal signal of the chip, and provides a more efficient development way for the high-density integration of the chip [2]. Semiconductor technology, thus, has entered the post-Moore era. However, at the same time, the heat generation rate per unit area of the 3D stacked chip has surged, which poses a more severe challenge to the chip thermal management technology [3,4].

Traditional air cooling, liquid cooling, heat pipe cooling, and semiconductor cooling struggle to meet the cooling needs of highly integrated chips. Therefore, many scholars have carried out extensive research on the thermal management of chips [5,6,7]. Among them, Tuckerman et al. [8] first proposed to construct tiny channels on the silicon base layer of the chip through chemical etching to allow the cooling fluid to pass directly, which can bypass the chip package and directly cool the surface of the integrated circuit. This embedded cooling channel provides a new solution for chip cooling [9].

Due to the broad application prospects of micro-channels, scholars have carried out extensive studies on the structure of micro-channels [10,11,12,13,14,15,16], heat transfer medium of micro-channels [17,18], heat transfer and flow properties of micro-channels [19,20,21], etc. In recent years, with the vigorous development of the new generation of artificial intelligence technology, machine learning and optimization algorithms have injected new vitality with intelligent characteristics into micro-channel research.

Bayer et al. [22] optimized the structure of a double-layer wavy wall micro-channel radiator based on a neural network, which greatly reduced the calculation time required for the optimization design compared with the traditional optimization method. Tafarroj et al. [23] established an artificial neural network model to predict the *Nu* number and heat transfer coefficient on the basis of experimental data of nano-fluid flow in the micro-channel radiator. Chen et al. [24] optimized the structure of the embedded micro-channel in the 3D disc model based on the NSGA-II algorithm. Wang et al. [25] optimized micro-channel heat sink structure with porous fins based on the NSGA-II algorithm, and the results showed that the overall best performance could be achieved by a tradeoff between the pumping power and the temperature drop.

At the same time, it is worth noting that the power level and spatial location of the thermal load of the practical chip are variable. As the chip’s operating tasks change, high-power regions are not fixed in certain positions [26,27,28]. Ansari et al. [29] analyzed the influence of the flow direction of cooling medium in double-layer micro-channels on the cooling performance under the condition of nonuniform heat sources with random distribution of hot spots. Li et al. [30] realized adaptive micro-channel cooling by using heat-sensitive nanocomposites. The heat-sensitive material expands when cooled but contracts when heated. Flow distribution can be adjusted according to the changing of heat load, and this provides more efficient and intelligent cooling for the areas with high heat flux density. Li et al. [31] applied a machine learning method, i.e., Bayesian optimization, to 3D integrated circuits with time-varying power distribution, realized intelligent control of coolant flow rate in micro-channels of specific layers, and kept the chip temperature stable within a safe range. However, no further research has been carried out on how to distribute the flow and enhance the cooling capacity of the channels.

It can be seen from the above that the thermal management adapting to the dynamic changes in thermal load (power) of the practical chip is very important and urgent, which has attracted the attention of many scholars. The development of intelligent technology provides an opportunity for the implementation of corresponding timely thermal management strategies. Therefore, this paper focuses on achieving highly efficient and timely intelligent cooling, when the power of 3D multi-core chips changes dynamically. A numerical simulation model of embedded liquid microchannels for cooling 3D multi-core chips is established, and an intelligent method combining a BP neural network and genetic algorithm is used to distribute the coolant flow rate. The method proposed in this paper can effectively reduce the chip temperature and improve the chip temperature uniformity without changing the channel structure. This paper could provide theoretical support for the realization of intelligent thermal management for 3D multi-core chips with high efficiency.

## 2. Model Building

### 2.1. Geometric Model

**Figure 1:**
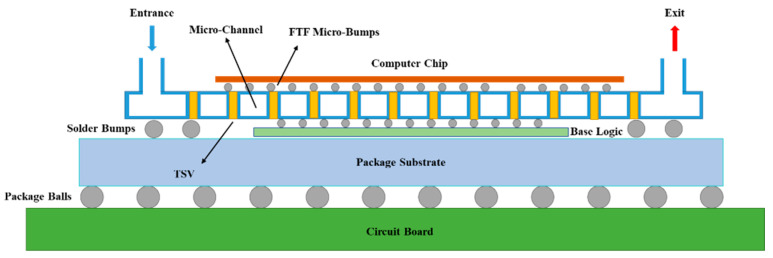
Schematic diagram of the 3D integrated chip structure with embedded cooling micro-channels.

Figure 1 presents a schematic diagram of a 3D integrated chip with embedded cooling channels. As shown in the figure, the upper and lower chips were connected by a passive adapter plate in the middle, and embedded cooling micro-channels were integrated in the middle of the passive adapter plate.

**Figure 2:**
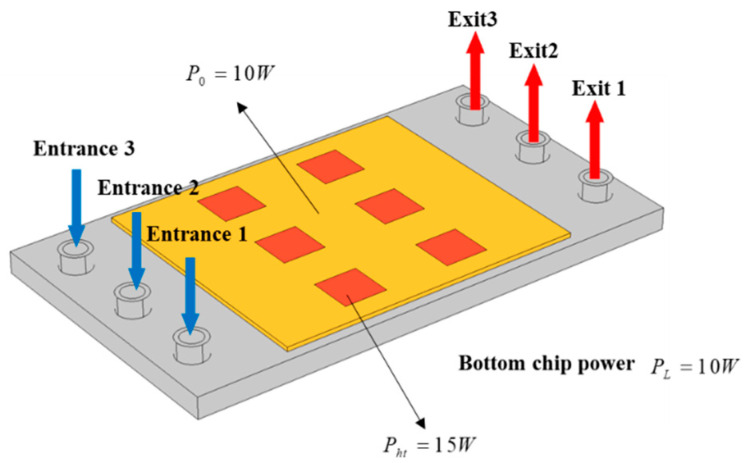
Schematic diagram of 3D model.

Figure 2 shows a schematic diagram of the 3D model. The liquid cooling channels had three inlets and three outlets. According to the change in the chip power distribution, the proportional flow rate of the three inlets could be adjusted to achieve the best cooling efficiency. The upper chip was a computing chip with six cores, each of which had a power of 15 W. The working state of each core was related to the task being performed by the chip. The lower chip was the basic logic chip, mainly used to realize basic functions, and its power was set at 10 W.

**Figure 3:**
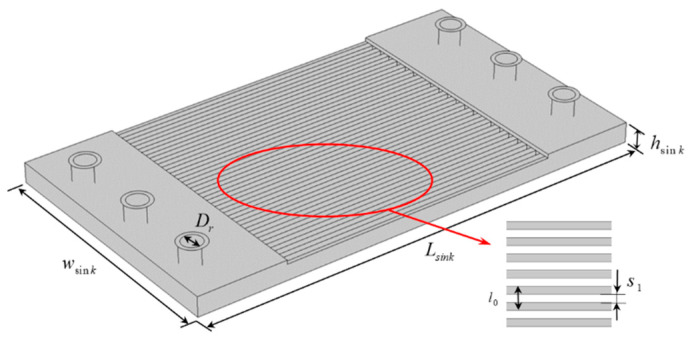
Geometry model of microchannel heat sink.

Figure 3 shows the dimensions of the microchannel heat sink [29]. The length, width, and height of the heat sink were 23,000 μm, 23,000 μm, and 1000 μm respectively. The wall thickness of the micro-channel was 20 μm. The number of channels was *N* = 21, and the diameter of the inlet/outlet was 700 μm.

### 2.2. Physical Model

In this paper, the solid material of the numerical model was silicon. The density, constant pressure heat capacity, and thermal conductivity of silicon were $\rho_{S}=2330[Kg\cdot m^{-3}]$, $c_{p,s}=712[J\cdot Kg^{-1}\cdot K^{-1}]$, and $k_{s}=148[W\cdot m^{-1}\cdot K^{-1}]$. The cooling medium was water, and the thermophysical parameters of water varied with temperature.

The simplified assumptions of the numerical model were as follows:

(1) The fluid flow and heat transfer were in a steady state, the cooling fluid was incompressible, and the flow state was laminar flow;
(2) The physical properties of solid material did not change with temperature, and the thermophysical parameters of the solid material were isotropic;
(3) The no-slip boundary condition was adopted for the walls;
(4) The model did not take into account the effects of gravity, the effects of radiative heat transfer, and the effects of heat dissipation due to fluid viscous dissipation.

On the basis of the above assumptions, the continuity equation is:

(1) $$\nabla \rho_{f}\cdot u=0.$$

The momentum conservation equation is:

(2) $$\rho_{f}\left(u\cdot \nabla u\right)=\nabla \cdot [-pI+\mu (\nabla u+{\left(\nabla u\right)}^{T})]+F.$$

The energy conservation equation for fluid is:

(3) $$u\cdot \nabla \rho_{f}c_{p,f}T_{f}=\nabla^{2}k_{f}T_{f}.$$

The energy conservation equation for solid is:

(4) $$\nabla^{2}T_{s}=0.$$

The energy equation for steady-state heat conduction of a constant heat source is:

(5) $$\nabla^{2}T+\frac{q^{\prime \prime }}{k_{s}}=0.$$

The continuity equation for the heat flux and temperature at the solid–fluid interface is:

(6) $$k_{s}\frac{\partial T_{s}}{\partial n}=k_{f}\frac{\partial T_{f}}{\partial n}.$$

(7) $$T_{s}=T_{f}.$$

In these formulas, $\rho_{f}$ is the fluid density, u is the fluid velocity vector, *p* (Pa) is the pressure, ***I*** is the unit matrix, F (N) is the body force vector, $k_{f}$ (W·m^−1^·K^−1^) is the thermal conductivity of the fluid, and $k_{s}$ (W·m^−1^·K^−1^) is the thermal conductivity of the solid.

The boundary conditions were as follows:

(1) The inlet water temperature was constant;
(2) The inlet mass flow rate was M_0_ = 0.001 kg/s;
(3) Outlet pressure was 0 Pa;
(4) The outer walls of the micro-channel were insulated, except where the micro-channel contacted the chip.

### 2.3. Model Validation

**Figure 4:**
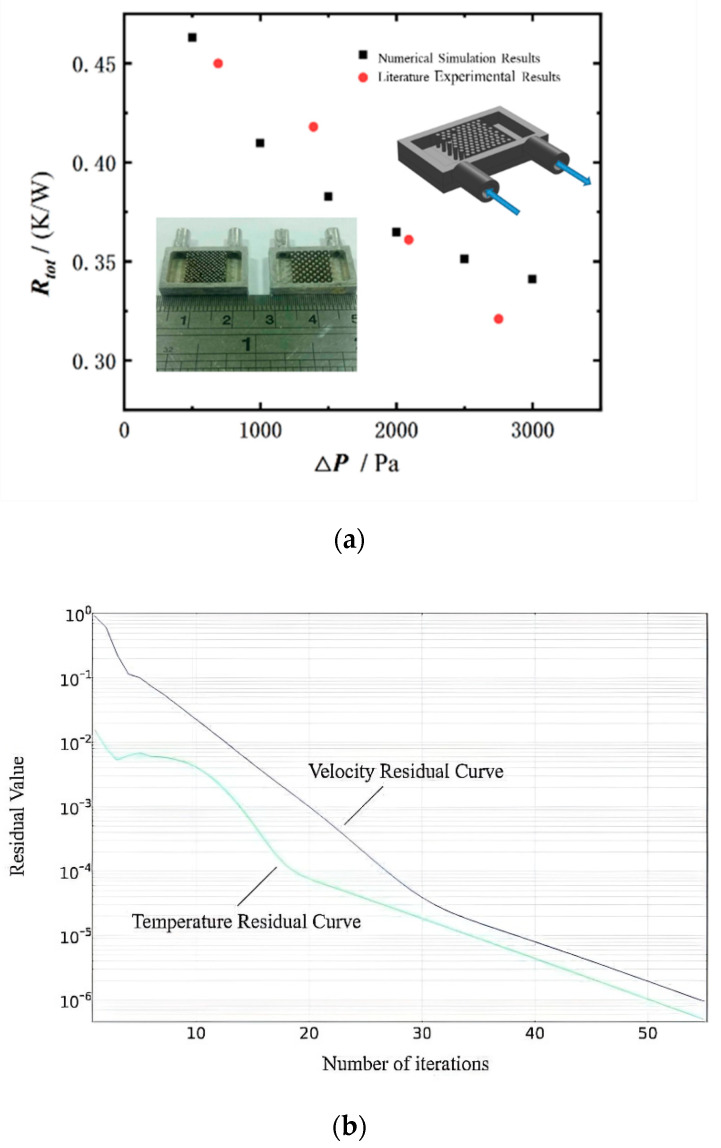
Model verification: (**a**) model reliability verification; (**b**) calculation residual curve.

COMSOL Multiphysics 5.6 was used to solve the governing equations with the corresponding boundary conditions. The grid numbers were 75,258, 278,271, 486,062, and 1,008,354, corresponding to 368.95 K, 371.38 K, 374.26 K, and 373.26 K respectively. The accuracy obtained on the basis of any numerical calculation model is directly related to the finite element mesh used. When more grids are used in a numerical calculation, its precision is higher, but more computing time and computing resources are required. In order to choose an appropriate number of grids, the relative error $\left|(T_{\max,i}-T_{\max,i-1})/T_{0}\right|$ of temperature was selected as the calculation criterion, where *T*_0_ is the corresponding temperature when the grid number was 1,008,354. The relative errors were 1.15%, 0.50% and 0.27%, respectively. Considering the time and accuracy of the examples in this paper, when the relative error was 0.27%, it was considered that the accuracy requirements were met. Thus, the meshing strategy of 486,062 was selected. In order to further verify the accuracy of the numerical model, the micro-needle fin heat sink model in [32] with forced convection heat transfer and sizes similar to those in the model shown in Figure 3, was established using the modeling method in this paper. Figure 4a presents a comparison of the simulation results with the experimental results in [32]. It can be seen from the figure that the maximum error between the thermal resistance of heat sink obtained using this method and the thermal resistance measured experimentally [32] was 0.071, while the minimum error was 0.011. Figure 4b shows that the calculation residuals for temperature and velocity converged to 10^−6^, i.e., the calculations can be considered to be converged.

## 3. Optimization Method

**Figure 5:**
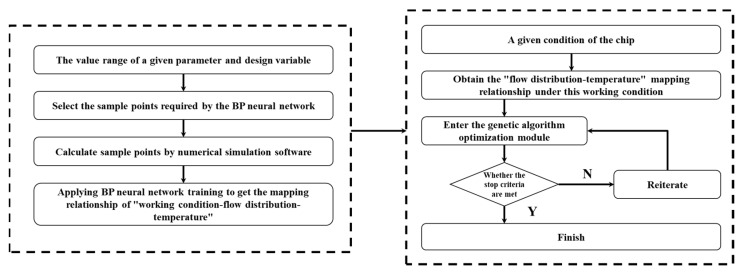
Flowchart of the optimization scheme.

Figure 5 presents the optimization flowchart. Steps 1–3 were used to obtain neural network training sample points, i.e., (1) determine the range of variables and parameters; (2) select sample points for neural network training; (3) obtain sample points required for BP neural network training through COMSOL numerical calculations. Steps 4–5 were used to obtain the “working condition–flow distribution–temperature” relationship of the model of embedded liquid microchannels for cooling 3D multi-core chips through a neural network, i.e., (4) perform function fitting on the sample points, and obtain “working condition–flow distribution–temperature” neural network model of embedded liquid microchannels for cooling 3D multi-core chips using the BP neural network algorithm; (5) according to the “working condition–flow distribution–temperature” mapping relationship, obtain the “flow distribution–temperature” mapping relationship under the given conditions. Step 6 was used to find the optimal flow distribution on the basis of the mapping relationship, i.e., (6) taking the “flow distribution–temperature” mapping relationship as the objective function of the genetic algorithm for seeking the minimum temperature, obtain the optimal flow distribution under a given chip working state.

**Figure 6:**
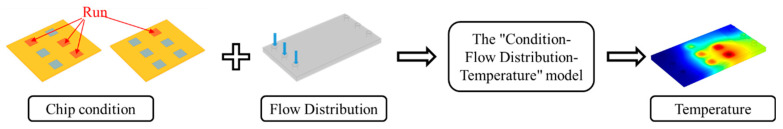
The legend of “condition–flow distribution–temperature” model.

Figure 6 shows the legend of the “condition–flow distribution–temperature” model. The “condition–flow distribution–temperature” model was a mapping relationship obtained through neural network fitting. The maximum temperature of the chip could be obtained after establishing the chip working condition and inlet flow distribution.

### 3.1. BP Neural Network Training

**Figure 7:**
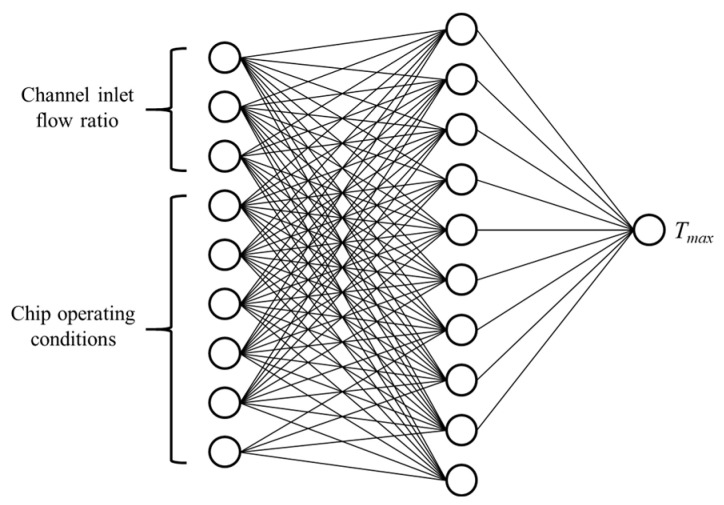
Schematic diagram of neural network configuration.

The computing module of the selected 3D chip consisted of six cores, and the working states of each core were independent of each other. The embedded liquid cooling micro-channel had three inlets, and the total flow rate was M_0_ = 0.001 kg/s. The BP neural network was trained on the basis of the sample points. The ratio of training data to testing data was 8:2. The hierarchical structure of the neural network was an input layer with nine artificial neuron cells, a hidden layer with 10 artificial neuron cells, and an output layer with one artificial neuron cell. Figure 7 shows a schematic diagram of the neural network configuration. The improved Bayesian regularization algorithm was used to train the established neural network.

**Figure 8:**
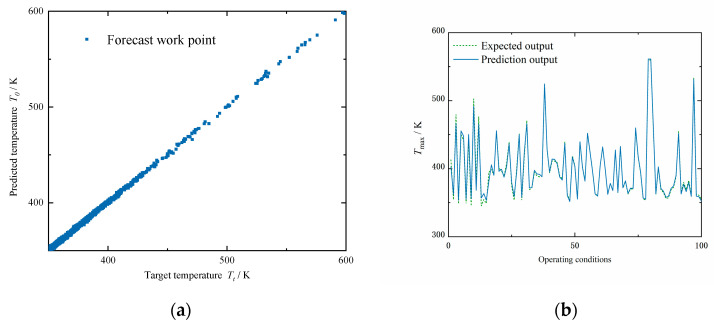
(**a**) Fitting diagram of BP neural network training results; (**b**) prediction output of BP neural network.

Figure 8a shows the scatter plot of the target output and the prediction output. The correlation coefficient between the target output and all 1494 samples was 0.99327. Figure 8b shows the comparison between the expected output and predicted output of the neural network under 100 different chip operating conditions in the test dataset.

### 3.2. Genetic Algorithm Optimization

For a given chip working condition, the mapping relationship between flow distribution and temperature was obtained using the neural network, and then the genetic algorithm was used to obtain the optimal flow rate distribution. Considering the accuracy requirements of this problem and the diversity of the generated individuals, the binary code number of the entry flow was 5 bits, the maximum genetic generation number was 500, the generation gap was 0.8, the crossover probability was 0.7, and the odd-numbered individuals were crossed with their adjacent offspring.

## 4. Result Analyses

Table 1 shows the working conditions for four different chips. Because of the different running programs of each chip, the working state of each chip core was different.

**Figure 9:**
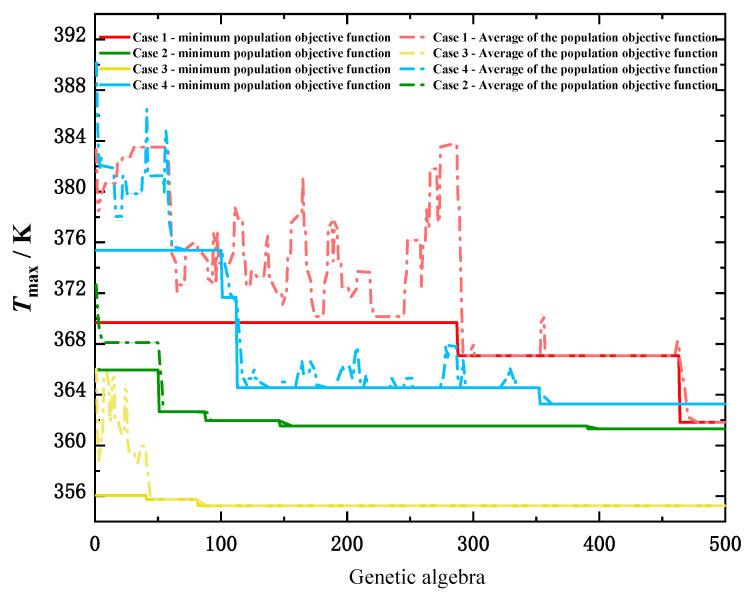
Intergenerational optimization trajectory diagram.

Figure 9 shows the intergenerational optimization trajectories for finding the optimal flow distribution through the genetic algorithm under four different chip working conditions.

Table 2 shows the comparison of the maximum operating temperature of the chip before and after the optimization of the flow distribution under the condition of a certain total flow. It can be seen from the table that, after the optimization of flow distribution, the maximum temperature corresponding to the chip was reduced. The maximum temperature of working condition 1 was decreased by 2.63 K, that of working condition 2 was decreased by 2.63 K, that of working condition 3 was decreased by 6.06 K, and that of working condition 4 was decreased by 4.63 K.

**Figure 10:**
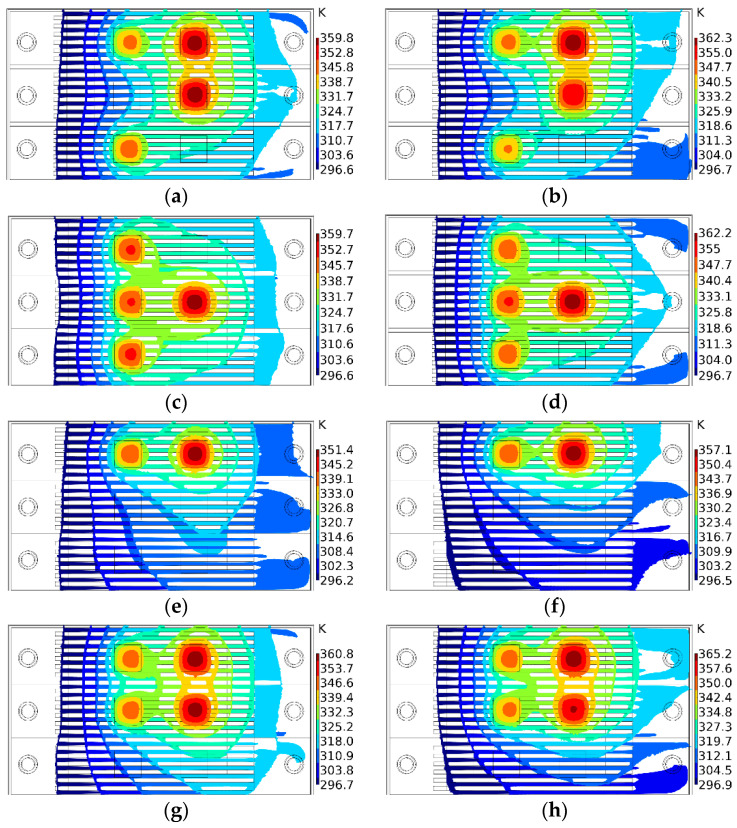
The isotherm diagrams corresponding to the chips before and after optimization. (**a**) Case 1 before optimization. (**b**) Case 1 after optimization. (**c**) Case 2 before optimization. (**d**) Case 2 after optimization. (**e**) Case 3 before optimization. (**f**) Case 3 after optimization. (**g**) Case 4 before optimization. (**h**) Case 4 after optimization.

Figure 10 shows the temperature contours of the inlet flow before (left) and after (right) optimization under the four chip conditions. It can be seen that, under the premise that the total flow remained unchanged, through the optimization of the genetic algorithm, increasing the distribution ratio of the flow to the operating area of the chip could reduce the maximum temperature of the chip during operation. At the same time, it can be found that the chip operating conditions corresponding to the optimized flow distribution had better temperature uniformity. This is because, when the flow was evenly distributed, the coolant over-cooled the nonworking areas of the chip, thereby increasing the temperature differential across the chip.

**Figure 11:**
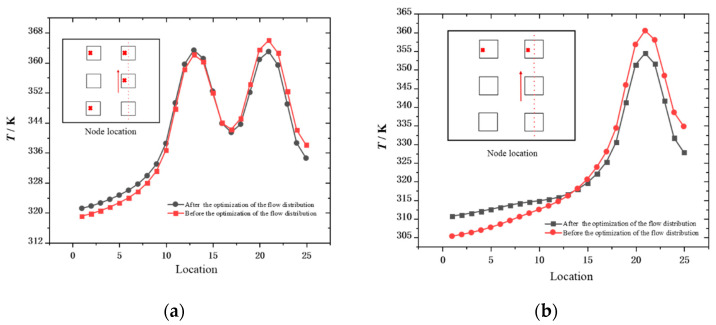
Changes in temperature at lateral nodes of the chip before and after flow optimization: (**a**) case 1; (**b**) case 3.

Figure 11a,b show the temperature variation of the chip lateral nodes before and after flow optimization. The running chip core in the picture is marked with a red cross. It can be seen from the figure that, after the flow distribution optimization, the maximum temperature of the chip operation was reduced. At the same time, the difference between the highest temperature and the lowest temperature on the chip surface was also reduced. The difference between the highest and lowest temperature in Figure 11a decreased from 46.86 K to 41.83 K. The difference between the highest and lowest temperature in Figure 11b decreased from 55.24 K to 43.78 K. This is due to the fact that the even distribution of the flow allowed the micro-channels to over-cool the nonoperating regions of the chip and under-cool the operating regions compared to the smart optimized flow distribution. The intelligently optimized flow distribution could more effectively cool the operating area of the chip, and it improved the temperature uniformity of the A surface. The uniform temperature distribution on the surface of the chip is beneficial to reduce the transfer delay of the signal inside the chip, while avoiding the thermal stress due to the excessive temperature gradient that causes local warping of the chip.

## 5. Conclusions

In this paper, a numerical simulation model of embedded liquid microchannels for cooling 3D multi-core chips was established. Aiming at the thermal management problem when the working power of the practical chip changes dynamically, the temperature field sample information was obtained by numerical calculation. The BP neural network was trained on the basis of the sample data to obtain the “working condition–flow distribution–temperature” mapping relationship. The optimal flow distribution strategy was further optimized using a genetic algorithm to adapt to the dynamic change of power, so as to minimize the working temperature of the chip under corresponding working conditions. Compared with the currently commonly used uniform flow distribution method, the intelligently optimized nonuniform flow distribution method further reduced the maximum temperature of the chip during operation and improved the uniformity of the chip temperature field. Under the given test conditions, the maximum temperature could be reduced by a maximum of 6.06 K, and the temperature difference on the chip surface could be reduced by a maximum of 11.46 K. It can be seen that the method developed in this paper can provide timely intelligent and efficient cooling for 3D multi-core chips under different working conditions.

## Figures and Tables

**Table 1:** Chip operating conditions.

Chip Condition	Core 1	Core 2	Core 3	Core 4	Core 5	Core 6
Case 1	Running	Not running	Not running	Running	Running	Running
Case 2	Running	Not running	Running	Running	Running	Not running
Case 3	Not running	Not running	Not running	Not running	Running	Running
Case 4	Not running	Not running	Running	Running	Running	Running

**Table 2:** Chip operating conditions.

Chip Condition	Flow Distribution	Maximum Temperature	Residual Value
Case 1 after optimization	29:29:42	363.38 K	2.63 K
Case 1 before optimization	1:1:1	366.01 K	
Case 2 after optimization	25:48:27	363.29 K	2.63 K
Case 2 before optimization	1:1:1	365.92 K	
Case 3 after optimization	14:29:57	354.5 K	6.06 K
Case 3 before optimization	1:1:1	360.56 K	
Case 4 after optimization	14:43:43	364.41 K	4.63 K
Case 4 before optimization	1:1:1	369.04 K	
